# Mutation rate analysis via parent–progeny sequencing of the perennial peach. I. A low rate in woody perennials and a higher mutagenicity in hybrids

**DOI:** 10.1098/rspb.2016.1016

**Published:** 2016-10-26

**Authors:** Zhengqing Xie, Long Wang, Lirong Wang, Zhiqiang Wang, Zhenhua Lu, Dacheng Tian, Sihai Yang, Laurence D. Hurst

**Affiliations:** 1 State Key Laboratory of Pharmaceutical Biotechnology, School of Life Sciences, Nanjing University, Nanjing 210023, People's Republic of China; 2 Chinese Academy of Agriculture Sciences, Zhengzhou Fruit Research Institute, Zhengzhou 450009, People's Republic of China; 3 The Milner Centre for Evolution, Department of Biology and Biochemistry, University of Bath, Bath BA2 7AY, UK

**Keywords:** peach, mutation rate, generation time, heterozygosity

## Abstract

Mutation rates vary between species, between strains within species and between regions within a genome. What are the determinants of these forms of variation? Here, via parent–offspring sequencing of the peach we ask whether (i) woody perennials tend to have lower per unit time mutation rates compared to annuals, and (ii) hybrid strains have high mutation rates. Between a leaf from a low heterozygosity individual, derived from an intraspecific cross, to a leaf of its selfed progeny, the mutation rate is 7.77 × 10^−9^ point mutations per bp per generation, similar to *Arabidopsis thaliana* (7.0–7.4 × 10^−9^ point mutations per bp per generation). This suggests a low per unit time mutation rate as the generation time is much longer in peach. This is supported by our estimate of 9.48 × 10^−9^ point mutations per bp per generation from a 200-year-old low heterozygosity peach to its progeny. From a more highly heterozygous individual derived from an interspecific cross to its selfed progeny, the mutation rate is 1.38 × 10^−8^ mutations per site per generation, consistent with raised rates in hybrids. Our data thus suggest that (i) peach has an approximately order of magnitude lower mutation rate per unit time than *Arabidopsis*, consistent with reports of low evolutionary rates in woody perennials, and (ii) hybridization may, indeed, be associated with increased mutation rates as considered over a century ago.

## 1. Introduction

Mutation rates vary between species, between strains within species [1–3] and between regions within a genome [4,5]. At these three levels, different predictors have been suggested as being relevant. In this paper, we focus on the possibilities that (i) woody perennials might have low mutation rates [6–8] compared with fast growing annuals, and (ii) hybrid strains have higher mutation rates [9]. In an accompanying paper, we ask whether recombination might be mutagenic [10,11] and whether the recombination rate is raised in this domesticated species [12–14].

### (a) Is hybridization mutagenic?

The hypothesis that hybrids/heterozygosity might be associated with increased mutation has a possibly surprisingly ancient pedigree. In 1915, Duncan [9] experimentally tested the hypothesis that inter-racial crosses might have high mutation rates. His inspiration was Darwin, who commented on the ‘notorious’, ‘extreme amount of variability’ in fertile hybrids and ‘mongrels’ (for quotes, see [9]). While Duncan found few mutations and considered the hypothesis thus unlikely, his analysis was influential. Indeed, what several more recent authors [15–17] now consider the seminal paper on the hybridization–mutation hypothesis, that of Sturtevant [18], was directly influenced by Duncan [9] and Belgovsky [19]. Both Sturtevant and Belgovsky found increased mutation rates in hybrids in flies. Intraspecific mating between populations [15,20] can also affect the mutation rate. The extent to which these effects are observed outside of flies (where the effect is probably owing to P element hybrid dysgenesis) is poorly resolved (but see e.g. Kostoff [21]), and no studies have examined this issue using next generation sequencing (NGS) considering between-species hybrids, although in *Arabidopsis* an effect in between eco-types crosses has been examined [4,15]. An NGS study would be valuable as it permits analysis of numerous phenotypically invisible mutations that have occurred over a very short time span, thus largely free of the filter of selection.

### (b) Why might hybrids have a higher mutation rate?

The problem of hybrid mutagenesis is intimately linked with the relationship between mutation and heterozygosity. While a language of heterozygote instability (originally proposed by Demerec [22], later independently proposed [23]) has been suggested, distinguishing between heterozygote-*associated* and heterozygote-*induced* mutation is of substance. Consider that the mutation rate is determined by a two-component complex, protein A and protein B, then we can imagine one species/strain at fixation for *A* and *B* alleles, and another at fixation for *a* and *b* alleles. The *AABB* × *aabb* cross generates individuals that can form AB, ab, Ab and aB protein complexes. If the latter two are not co-adapted, then an increase in the mutation rate might result [18]. As homozygous *AAbb* and *aaBB* individuals would also have raised mutation rates, heterozygosity is not causative. The effect is, however, observed in heterozygous hybrids and so may be considered heterozygous-*associated*. Hybrids may also be in a state of low viability or stress and, via mechanisms unknown, stress can induce raised mutation rate (for review see [24]).

Heterozygosity might also directly induce raised mutation rates. A pan-genomic mutation rate effect, owing to heterozygosity at one particular locus, could, for example, reflect the product of a protein homodimer in which heterozygotes are less effective. Woodruff *et al*. [25] consider such a model, reporting released mutator activity in *Drosophila* hybrids and conjecture a role for heterozygosity of suppressor alleles. They also consider that there may be alleles at multiple loci, arguing that no two populations need involve the same loci in mutation rate suppression. An alternative possibility is that local DNA-based effects might modulate local mutation rate variation with heterozygotes being more locally mutable (for possible mechanisms, see [23] and [4]). This possibility was considered in the pre-sequencing era, but largely rejected. Emerson [26, p. 510], however, reports ‘*Mutations from variegation to self color occur more frequently in the heterozygous, V W, than in the homozygous, V V*’*.* He may have been confusing organelle segregation with mutation. Nonetheless, Demerec, inspired by this result, examined whether the miniature gamma 3 gene in flies [22] and Rose-alpha gene of *Delphinium* [27] are more mutable in the heterozygous condition. His results are largely negative. He notices [22, p. 658] in passing, however, that ‘*In case of the unstable reddish, … it becomes unstable in females only when it is in the heterozygous condition*’.

Much other evidence is negative or supportive of alternative models. Demerec ([28]), for example, demonstrated variable mutation rates between strains in *Drosophila's* miniature gene and tracked via linkage analysis many mutation rate modifiers. These were unlinked to the gene in question, suggesting global, not local, modifiers. Timofeef-Ressovsky [29], interested in whether heterozygosity affects the mutation rate, introgressed a white allele from a Russian population of flies into an American population. She finds no heterozygosity effect. Further evidence against the local heterozygosity model comes from observations that the raised hybrid mutation rates are seen in haploid parts of the genome [16,20] (i.e. the X), and that the effects are commonly reported to be dependent on the direction of the cross [15,16,18]. This might be expected were there X-autosome interactions of a co-adapted gene complex. Furthermore, the extent of the effect can be stronger in mating between more closely related populations than more distant ones, so unlikely to correlate with heterozygosity in a linear manner [15].

With the introduction of sequencing, some authors have advocated that many intragenomic correlations are consistent with heterozygosity having local effects on mutation (for review, see [23]; see also [30,31]). These correlations provide indirect inferences [23] and cannot disentangle cause and effect [23]. More parsimonious explanations are commonly possible. Indeed, the simplest null hypothesis supposes that a higher mutation rate would correlate with higher heterozygosity, all else being equal, this being a prediction of the neutral theory. A higher heterozygosity seen in African human populations should then be most acutely seen for classes of site with higher mutation rates , as observed in [32]. While single nucleotide polymorphism (SNP) clustering (see e.g. [33]) has been interpreted [30] as consistent with local heterozygosity-induced mutations, many forces affect regional mutation rates, on many different scales [5,34], and provide alternative explanations.

Most problematic for interpretation of correlation-based results is biased gene conversion. This process increases the frequency, but not the mutation rate, of GC residues at GC–AT mismatch sites in an allelic hetero-duplex [35]. Intra-locus biased gene conversion requires heterozygosity to operate, but its effects are easily mistaken for mutation rate changes [36]. The correlation between substitution rate at putatively neutral sites and recombination [37]/heterozygosity, cited as consistent with the local heterozygosity instability hypothesis [23], is more parsimoniously explained by biased gene conversion [36]. Biased gene conversion has modulated genetic distances and branch lengths between human populations [38]. The observation of higher divergence from chimpanzee in more heterozygous human populations (Africans versus non-Africans) [31] was advocated as evidence for heterozygous mutational instability [31], thus may well have a simpler explanation.

While the possibility that localized heterozygosity causes increases in the local mutation rate is not convincingly supported (at least for point mutations), an observation of increased mutation rates in genomic sub-compartments made to be heterozygous [4], and thus in proximity to extant heterozygous sites, is suggestive. Here, we attempt to replicate part of this analysis in a different species. To this end, we ask both whether a heterozygous F_1_ has a higher mutation rate than one with lesser heterozygosity and whether new mutations tend to occur in proximity to heterozygous sites. If they occur randomly, this would not support a local heterozygosity model. If they do not occur at a higher rate, this would not support any model of heterozygous-instability (heterozygote associated or induced mutation).

We consider the mutation rate in the domesticated peach. Although largely selfing [39], self-fertile interspecific hybrids are viable. While peach has extensive linkage disequilibrium, it is unclear why this or other features might interfere with mutation rate estimation. The current best practice for mutation rate estimation is to employ parent–offspring comparisons via high-quality, high-stringency whole-genome sequencing. The direct estimation approach avoids the problem of misinference owing to biased gene conversion, and requires no assumption of effective neutrality. Indeed, in many lineages, all synonymous mutations (commonly employed as putatively neutral sites) cannot be assumed to be effectively neutral [40,41]. Peach has a notable disadvantage in that it has a relatively long generation time, this being no less than 3 years [42].

## 2. Material and methods

### (a) Sampling

We analysed three parent–progeny groups (groups I ∼ III). Each has an F_1_ parent tree together with its selfed F_2_ progeny. The F_1_ parent trees were derived from crosses either between different peach cultivars, or between different *Prunus* species. Groups I and II are intraspecific low heterozygosity crosses, employing young (group I) and old (group II) F_1_s, while group III F_1_ is an interspecific cross. The older parent we employ as a check on the possible effects of somatic mutations and to confirm the effects of low intraspecific heterozygosity. Group I included one weakly heterozygous F_1_ (*Prunus persica*) and 24 selfed F_2_ samples (144F2-1 to -24 in table 1). Group II included one weakly heterozygous F_1_ (*Prunus mira*, a wild peach) and nine selfed F_2_ samples (GZTH-S1 to GZTH-S5, GZTH-S7 to GZTH-S9 and GZTH-5). The interspecific crossing group (group III) (electronic supplementary material, figure S1) included four ancestral parents, one heterozygous F_1_ (*Prunus davidiana* × *P. persica*) and 30 F_2_ samples, the selfed progeny of the F_1_ (NE1-NE30 in table 1). In total, 70 peach samples, including four ancestral parents from group III, three F_1_ parents (i.e. each group with one F_1_ sample), and 63 F_2_s, were selected for whole-genome resequencing. The average nucleotide diversity (number of nucleotide differences per site) was approximately 0.29%, 0.27% and 1.24% at the whole-genome level between the two haplotypes derived from a single F_1_ in groups I, II and III, respectively. For further details on sampling and handling, see the electronic supplementary material, methods and figures S1 and S2.

**Table 1.** Number of spontaneous mutations per generation in the peach genome. (Summary statistics are given in italics.)

samples	SNPs	indels	samples	SNPs	indels
intraspecific groups					
144F2-1	3	0	144F2-18	4	2
144F2-2	2	0	144F2-19	4	2
144F2-3	2	0	144F2-20	2	1
144F2-4	1	0	144F2-21	5	0
144F2-5	5	0	144F2-22	1	0
144F2-6	4	1	144F2-23	3	1
144F2-7	0	0	144F2-24	3	1
144F2-8	6	1	GZTH-5	4	4
144F2-9	3	0	GZTH-S1	2	1
144F2-10	2	1	GZTH-S2	1	0
144F2-11	0	0	GZTH-S3	4	0
144F2-12	2	0	GZTH-S4	5	0
144F2-13	4	0	GZTH-S5	4	1
144F2-14	3	0	GZTH-S7	1	0
144F2-15	3	1	GZTH-S8	2	1
144F2-16	4	0	GZTH-S9	2	0
144F2-17	5	0	*mean* (±*s.e.*)	*2.91 ± 0.27*	*0.54 ± 0.15*
interspecific group					
NE1	5	0	NE17	3	1
NE2	8	2	NE18	4	0
NE3	3	1	NE19	7	2
NE4	12	1	NE20	7	1
NE5	4	1	NE21	11	2
NE6	3	2	NE22	3	0
NE7	4	1	NE23	5	0
NE8	4	0	NE24	3	0
NE9	6	1	NE25	4	0
NE10	6	2	NE26	6	0
NE11	4	1	NE27	3	2
NE12	7	2	NE28	4	2
NE13	5	1	NE29	4	0
NE14	0	0	NE30	3	1
NE15	4	1	*mean* (*±s.e.*)	*4.80 ± 0.45*	*0.93 ± 0.14*
NE16	2	1	** **		

### (b) Sequencing and alignment

Fresh leaves were collected from each plant, and stored at −80°C. DNA was extracted using cetyl trimethylammonium bromide method [43]. For two samples GZTH-5 and GZTH-8, the DNA was directly extracted from the seed after careful removal of the seed coat. All samples were sequenced using 150 bp paired-end Illumina Hiseq4000 platform at the Beijing Genomics Institute, with a library insert size of 350 bp. Each sample was sequenced to at least 40× (electronic supplementary material, table S1). Raw reads were cleaned by removing adaptors and low-quality reads, ensuring over 95% of the clean data have a base quality more than or equal to 20 (e.g. Q20 ≥ 95%).

The high-quality whole-genome shotgun assembly of peach cv. Lovell was used as the reference genome [44] (download of Peach v. 2.0 from https://www.rosaceae.org/species/prunus_persica/genome_v2.0.a1). Cleaned reads were mapped to the reference using BWA-mem 0.7.10-r789 [45] with option ‘-M’, the results were written to bam files. Bam files were processed with Picard tools MarkDuplicates v. 1.114 to mark PCR duplicates, followed by local realignments around putative indel loci using RealignerTargetCreator and IndelRealigner in GATK package v. 3.3.0 [46].

### (c) Variant calling

Initial variants for each sample were called using GATK HaplotypeCaller (HC) and UnifiedGenotyper (UG) [46]. The HC was run in the GVCF mode for each sample with default parameters, followed by combined genotyping across all samples within the same group. By default, HC requires a minimum mapping quality of 20 to generate confident calls. The UG was running with parameters ‘-glm BOTH -rf BadCigar -rf MappingQuality -mmq 20′, which requires a minimum mapping quality of 20. Raw variant calls were directly analysed without further filtering, as more pre-filtering steps would lead to a higher false-negative rate.

To generate a high-confidence variant set, we use only bi-allelic variant loci with (i) quality more than or equal to 50, (ii) a depth no less than 10 and not exceeding 80, and (iii) more than half of samples contain informative calls in each group. To reduce genotyping errors, we also required a reference-allelic ratio of 0 ∼ 5% or 95 ∼ 100% to call a homozygote, while 30 ∼ 70% was required to call a heterozygote. A confident marker was thus identified where the F_1_ samples were present in a confident heterozygous status. Mapping errors owing to highly similar paralogous sequences could also result in pseudo-heterozygosity. To minimize these errors, we remove those markers residing in large structural variant (SV) regions of F_1_ samples compared with the reference genome in each group (see the electronic supplementary material, methods).

### (d) De novo mutation identification

The candidate mutations were identified by searching for mutation alleles present in a single progeny only and not in the parent or other progeny of the same parent. To detect mutations, we use a pipeline previously described [4] with slight modifications. The approach has a negligible false-positive discovery rate and a circa 10% false-negative rate [4]. We modified the detection pipeline in order to minimize any possible false negatives (FN) owing to variant callers. Therefore, we further applied the following procedures to both variant sets from HC and UG.

Genotyping errors in non-mutated samples could cause a failure to detect a true mutation with the same genotype called. To address this, we started from the rare variants with a frequency of less than three in each group as the initial candidates. For all SNP candidates in each sample, we counted the covered reads for all present alleles in each strand using VarScan (v. 2.3.6) readcounts [47]; for indel candidates, we regenerated those indel calls by running HC in a joint-calling model, from which a more accurate allele depth was obtained for each sample (present in allelic depth field in generated VCF file). By directly comparing the reads covered upon each sample, we purged genotyping errors and were able to efficiently remove false positives, under the premise that reads from sequencing or mapping artefacts were less likely to be shown only in a single sample.

Candidate mutations were detected by requiring: (i) at least five reads with both forward and reverse strands in the focal sample (e.g. the sample carries a different allele from all other samples), (ii) the parental samples should contain informative calls as a background, and no more than five ‘missing’ data calls in other F_2_ samples (a high ‘missing’ rate in each group is also a sign of low variant quality), and (iii) no evidence that the same mutationally derived allele is present in either parental samples or other F_2_ progeny. All processed loci failing previous criteria were soft-masked (instead of direct hard filtering), and only loci passing all criteria were marked as ‘PASS’. We also masked loci with a clustering status (defined as more than three base substitutions within 10 bp or more than two indels within 20 bp) as those loci are most likely owing to contamination.

Afterwards, all ‘PASS’ candidates were manually investigated. The integrative genomics viewer (IGV) [48] was applied to review the mapping states across all samples within the candidate loci. We also extracted all aligned reads for each candidate locus from each sample, and realigned those reads to the reference sequence with ClustalW2 [49] to get a more accurate alignment, and then manually inspect each alignment in combination with IGV. Candidate loci resulted from spurious mapping artefacts or possible contamination (detected by BLAST search in the NCBI Nucleotide collection database using the aligned reads) were discarded. Masked loci failing previous criteria were randomly sampled, and also manually reviewed to make sure no true mutation was filtered out.

The final mutation results were obtained by combining all passed candidates from both UG and HC sets. Most mutations were detected by both variant callers. The consistency rate is higher for point mutations (213 of 240, 88.8%) than for indels (32 of 46, 69.6%). The HC performs better in indel detection owing to a local re-assembly algorithm, and 11 indel mutations were exclusively called from HC, while only 3 were exclusively called from UG. For base substitution, UG missed 16 calls detected by HC, while HC lost 11 calls predicted by UG. A soft-masking strategy was effective in controlling the FN and helps in adjusting the filtering criteria to obtain the best possible results. The detection pipeline (starting from the raw variant sets) as well as accompanying scripts is available at https://github.com/wl13/BioPipelines/tree/master/Mutation_Detection.

### (e) Sanger validation of mutation calls

We designed PCR primers for 101 randomly selected point mutations and 25 indel mutations, followed by Sanger sequencing to confirm those mutation calls. For each mutation locus, the F_2_ sample, where this mutation was called, the F_1_ generation parental sample, and at least one additional F_2_ sample not supposed to carry the mutation were sequenced. Only mutation alleles verified in the called samples and absent in both parental samples and other F_2_ samples were considered as confirmed. Mutation loci failing to give valid results owing to PCR difficulties or poor sequencing results were considered as undetermined.

### (f) Estimation of mutation rate

The per generation per site mutation rate was calculated by dividing the average number of called mutations by twice the accessible haploid reference genome size. The accessible reference genome size (i.e. callable sites) was estimated using a simulation approach described in Keightley *et al.*, 2015 [50] (see the electronic supplementary material, methods and table S2). The overall false-negative rate within callable sites was estimated to be low (less than 1%).

### (g) Estimation of heterozygosity

For F_1_ samples in each group, the genome heterozygosity was estimated as the rate of heterozygous SNPs among all callable sites. This was done by genotyping each F_1_ sample using GenotypeGVCFs ‘--includeNonVariantSites’ option. For a confident heterozygous SNP, we require a minimum depth of 10 and a maximum depth of 80. We also calculated the reference-allelic ratio, defined as proportion of reference-allelic reads to the total covered reads. Only SNPs with a reference-allelic ratio between 30% and 70% were considered as a confident heterozygous call, while allelic ratios below 5% or above 95% were considered as a confident homozygous call. The same criteria were applied to all non-variant sites. The overall heterozygosity was estimated as number of heterozygous SNPs/(number of heterozygous SNPs + number of homozygous sites).

### (h) Statistical analysis

Statistics were performed in R [51]. A Brunner-Munzel (BM) test was implemented in R package ‘lawstat’. The trinucleotide content of point mutations was counted with the mutation at the start, centre and end of the triplet, and the mutation rate per given trinucleotide triplet was then calculated. The genome-wide trinucleotide content as well as triplets within heterozygous or homozygous compartments was also counted from the first, second and third nucleotide of each sequence. For each compartment, the expected number of point mutations was derived from the observed triplet mutation rate. Population diversity was calculated as the average pairwise differences among all possible pairs (electronic supplementary material, Methods). To estimate confidence intervals of the estimated mutation rate, we assume the number of mutations is a Poisson variable. We then apply the Poisson test function in R to estimate 95% confidence intervals.

## 3. Results

### (a) Mutation calling has no observable false-positive rate

In total, 240 base mutations and 46 small indel mutations were detected in the 63 F_2_s from three selfed F_1_ individuals (table 1; electronic supplementary material, table S3). To assess reliability, 101 base mutations and 25 indels were selected for verification by Sanger sequencing. Sanger sequencing confirmed that 100% of these sampled mutations were present in focal individual F_2_s, but absent in corresponding genomes of the F_1_ and other F_2_ samples.

### (b) No evidence that selection distorts the observed mutational profile

Analysis of the intragenomic location of new mutations suggests that purifying selection is not an important contributor to observed patterns. The interspecific group contained 23 mutations in coding regions and 149 mutations in non-coding regions (table 2), which was no different from the genomic expectation given relative proportion of coding and non-coding sequence (*χ*^2^_1_ with Yates correction = 0.146, *p* = 0.702). The intraspecific groups were slightly biased toward non-coding regions with only nine mutations in coding versus 105 mutations in non-coding regions (*χ*^2^_1_ with Yates correction = 3.679, *p* = 0.0551). Both groups had an excess of non-synonymous changes upon synonymous changes, which was not significantly different from the null mutational expectation of circa 3 : 1 (*χ*^2^_1_ with Yates correction = 0.167, *p* = 0.683 for intraspecific group; and *χ*^2^_1_ with Yates correction = 0, *p* = 1 for interspecific group).

**Table 2.** De novo mutations in coding and non-coding regions.

items	intraspecific groups		interspecific group	
	SNPs	indels	SNPs	indels
non-coding	88	17	126	23
coding	8	1	18	5
synonymous	1	—	4	—
non-synonymous	7	—	14	—
frame shift	—	1	—	4
non-frame shift	—	0	—	1

The absence of selection on de novo mutations was also inferred from the frameshift mutations, under the expectation that selection should skew towards an intragenic multiple of three indels. Of all 46 detected indel mutations, 40 of them, including 35 outside and five inside the coding regions, were not multiples of three bases long; the remaining six, including five outside and one inside the coding regions, are multiples of three. We find no evidence for an excess of multiples of three in coding sequences (Fisher's exact test, one-tailed *p* = 0.59).

### (c) Mutation is AT biased

**Figure 1.**
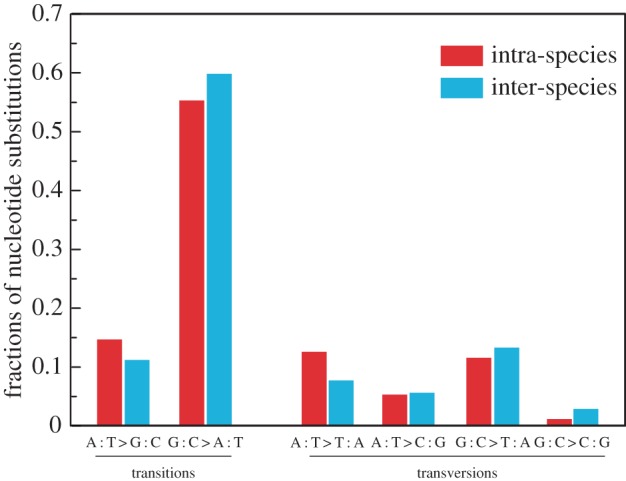
Mutation spectrum of intraspecific and interspecific groups. (Online version in colour.)

The 240 base mutations showed a transition–transversion bias and a GC->AT bias (table 3 and figure 1). Raw counts of GC->AT mutations indicate an absolute excess of GC->AT even though A : T and G : C compositions were 62.5% and 37.5%, respectively [52]. Correcting for nucleotide content, AT-biased mutations (G/C → A/T per GC) had 6.31-fold higher mutation rates than mutations in the opposite direction in intraspecific samples (A/T->G/C per A/T) and 8.96-fold higher mutation rates in interspecific samples. The highest proportion of mutations (per class of site) was from CpG sites after correcting for genomic background (electronic supplementary material, table S4), consistent with the presence of methylation in peach [53]. The transition/transversion (Ti/Tv) ratio is 4.76 (table 3), which is larger than inferred from substitutional analysis (3.2–3.6) [52]. One possible explanation is that GC-biased gene conversion opposing the mutation bias, may play a role in maintaining GC content in peach.

**Table 3.** Spectra of the base mutations. (Note that in the peach genome, the actual A : T and G : C compositions are 62.5% and 37.5%, respectively. Ti/Tv is a ratio of rates, not of observed events. As transitions are two times more frequent than transversions, the Ti/Tv ratio is twice the ratio of events = 2(Ti events/Tv events) = 4.76, which is larger than the population data (3.2–3.6).)

	intraspecific groups		interspecific group	
	number	fraction	number	fraction
type of mutations	** **	** **	** **	** **
transitions (total)	67	0.698	102	0.708
A : T > G : C	14	0.146	16	0.111
G : C > A : T	53	0.552	86	0.597
transversions (total)	29	0.302	42	0.292
A : T > T : A	12	0.125	11	0.076
A : T > C : G	5	0.052	8	0.056
G : C > T : A	11	0.115	19	0.132
G : C > C : G	1	0.010	4	0.028
A : T sites	31	0.323	35	0.243
G : C sites	65	0.677	108	0.757
total	96		144	

### (d) Peach has approximately the same per generation per site mutation rate as *Arabidopsis*

In the 286 de novo mutations, a total of 71, 25 and 144 base mutations (2.96, 2.78 and 4.80 on average) and 11, 7 and 28 indels (0.46, 0.78 and 0.93 on average) were detected in the parent–progeny groups I, II and III, respectively (table 1). We corrected the mutation rate of each group for their effective covered regions, which were 84.3%, 64.9% and 76.9% for groups I, II and III, respectively (electronic supplementary material, table S2). Thus, the final estimated de novo mutation rate for intraspecific crosses is 8.16 × 10^−9^ (95% confidence interval = 6.61 × 10^−9^–9.96 × 10^−9^) per site per generation. For indels, we observe 1.53 × 10^−9^ (95% confidence interval = 9.06 × 10^−10^–2.42 × 10^−9^) per generation per site. The indel rate is thus approximately one-fifth the point mutation rate, in line with prior direct sequencing approaches [4]. Group I have a younger parent and are thus possibly more representative of the time-averaged rate. These have a rate of 7.77 × 10^−9^, 95% confidence interval = 6.07 × 10^−9^–9.81 × 10^−9^.

It is striking that this intraspecific rate is comparable, on a per generation basis, to that seen in *Arabidopsis thaliana* (estimate 7.0 to 7.4 × 10^−9^ [4,54]). If we consider that peach has an approximately 10–20 times longer generation than *Arabidopsis*, this then equates to an approximately order of magnitude difference in the mutation rate per unit time, peach mutating much slower. This comes, however, with the caveat that the method, in requiring a mutation to be visible in one offspring alone, probably excludes some somatic mutations that occurred in the parent (but see below).

### (e) Hybrid individuals may have higher mutation rates

Should hybridization be predictive of the mutation rate then we expect interspecific crosses to have higher rates of mutation. In interspecific crosses, we observe a point rate of 1.38 × 10^−8^ (95% confidence interval = 1.17 × 10^−8^–1.63 × 10^−8^) for base mutations and 2.69 × 10^−9^ (95% confidence interval = 1.79 × 10^−9^–3.89 × 10^−9^) indel mutations, respectively. Thus, an approximately 1.8-fold (for base) and 1.76-fold (for indels) higher mutation rates were observed in interspecific groups compared with intraspecific group I (with an equally old parent), which is consistent with the prediction that hybridization is associated with higher mutation rates (BM test, *p* = 2.22 × 10^−5^ for base mutations and *p* = 0.0064 for indel mutations). The point to indel ratio remains almost unchanged at 1 : 5.

A possible explanation for the apparent increase in the mutation rate seen in interspecifics is that ‘mutations’ can be more easily called in heterozygous than in homozygous regions owing to artefacts. However, such an explanation should lead to two predictions: (i) more heterozygous regions should be present in F_2_s from the interspecific crossing group than from the intraspecific crossing groups, or (ii) more mutations should be detected in F_2_ heterozygous regions than in F_2_ homozygous regions, when all regions share the same F_1_ heterozygous background.

In contrast with the first prediction, a similar heterozygosis rate was found in the F_2_s of the interspecific crossing group (52.6%) and the intraspecific crossing groups (51.0%) (*t*-test, *p* = 0.62). Regarding the second prediction, of 254 mutations in intraspecific group I and interspecific group III whose backgrounds could be clearly assigned, 121 mutations were found in heterozygous regions, while 133 were found in homozygous regions of these F_2_ samples, which is not different from the null expectation (131.7 and 122.3 mutations expected for heterozygous and homozygous domains, respectively, *χ*^2^_1_ with Yates correction = 1.64, *p* = 0.20). The result holds after control for trinucleotide content (*χ*^2^_1_ with Yates correction = 1.18, *p* = 0.28), within the interspecific group (*χ*^2^_1_ with Yates correction = 0.75, *p* = 0.38; with correction for GC content; electronic supplementary material, figure S3A) and within the intraspecific group I (*χ*^2^_1_ with Yates correction = 0.40, *p* = 0.53; with correction for GC content, electronic supplementary material, figure S3B). Therefore, possible ease of calling artefacts could not explain the apparent higher mutation rate in the interspecific crossing group.

An alternative possibility is that if *P. davidiana* had a much higher mutation rate than *P. persica*, it may contribute to the higher mutation rate in interspecific groups independent of hybrid effects. In the interspecific F_2_ samples, 63 mutations were found in homozygous domains derived from *P. persica*, while 26 mutations were found in homozygous *P. davidiana* domains. After correcting for trinucleotide content and for the extent of unique covered regions in *P. persica* (93.5%) and *P. davidiana* (79.5%), mutation rates are not significantly different between the two haplotypes (55.5 base substitution from homozygous *P. persica* and 27.7 from *P. davidiana* after correcting for coverage, expected 48.9 and 34.3, respectively, *χ*^2^_1_ with Yates correction = 1.89, *p* = 0.170). Therefore, the data do not support the hypothesis that haplotypes from *P. davidiana* had a much higher mutation rate.

**Figure 2.**
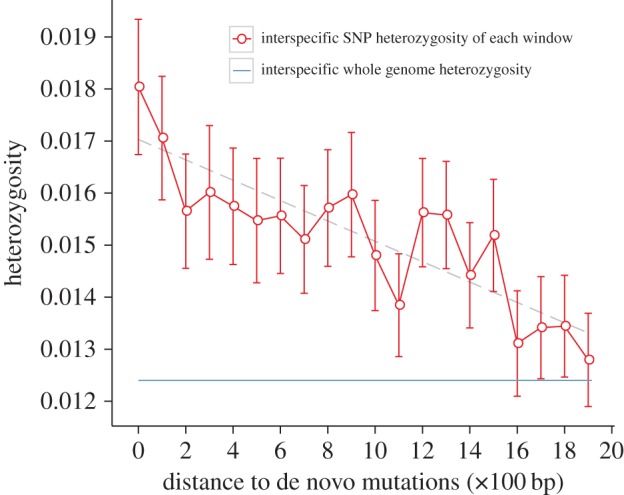
Relationship between interspecific SNP heterozygosity and the distance to de novo mutations. Window 0 in *x*-axis is 2×100 bp sequence surrounding the position of any given de novo mutation, and 1–19 is 100–1900 bp away from the mutation on both sides. For each window of 2×100 bp sequence, the SNP heterozygosity is calculated as described in the Material and methods section. Windows with fewer than 80 bp of informative sites were discarded. The red circles denote the SNP heterozygosity of the interspecific F_1_ sample, the blue line is the average genomic heterozygosity (0.0124) estimated for the interspecific F_1_ sample. Error bars, mean ± s.e. of the mean. The dashed line represents standard linear regression, and is for illustrative purposes only. (Online version in colour.)

Two models could predict higher mutagenicity in heterozygotes: first, a genome-wide effect that causes global increase in the mutation rate or second, a regionalized effect whereby proximity to a heterozygous site is predictive. If the second model is incorrect, we expect that de novo mutations should not be close to the heterozygous sites between the two haplotypes of the F_1_. However, the level of diversity surrounding mutation sites was higher than the genome average (1.24% between the two haplotypes of F_1_) (figure 2). Thus, we cannot reject the hypothesis that local heterozygosis between the two haplotypes might be causative.

### (f) Could somatic mutation provide a possible explanation for the difference between interspecific and intraspecific crosses?

The above results are in line with what has been previously observed in within and between ecotype crosses in *Arabidopsis*, in which between ecotype crosses have a higher net mutation rate [4,15]. However, unlike in *Arabidopsis*, a further potential difficulty stems from the fact that the interspecific F_2_ samples were a little older (approx. 3 years) than the intraspecific ones (about three months). The higher mutation rate in the interspecific group might thus come from the accumulation of more somatic mutations during its growth. To address this possibility, we consider two approaches.

First, we consider the mutation rate from a 200-year-old parent to 3-month-old F_2_ in *P. mira* (group II). If somatic mutation is of major consequence, then the mutation rate (F_1_ leaf to F_2_ leaf) in this cross should be considerably raised compared with the group I intraspecific F_1_ to F_2_, the F_1_ being less than a decade old, the F_2_s in both group I and group II samples being about three months. The logic here is that somatic mutations on any branch in the ancient F_1_ that was unique to any of our fruit will be called new mutations when comparing a leaf on another branch in the F_1_ with a leaf in the F_2_. This mutation would also not appear in the F_2_ siblings derived from different branches. We find the intraspecific *P. mira* F_1_ has a mutation rate (9.48 × 10^−9^, 95% confidence interval = 6.14 × 10^−9^–1.4 × 10^−8^) comparable to the group I intraspecific cross (7.77 × 10^−9^, 95% confidence interval = 6.07 × 10^−9^–9.81 × 10^−9^) with a much younger parent. However, the upper bounds (1.4 × 10^−8^) also just include our estimation for the interspecific hybrid (1.38 × 10^−8^).

Second, we can relax the requirement that to call a mutation in an F_2_, it must be observed in one F_2_ uniquely. While this assumption reduces the false-positive rate, it also excludes bona fide somatic mutations that occurred in the parent and were transmitted to multiple progeny. If somatic mutations are important, then they should be observed through multiple F_2_s. We thus searched for point mutations present in two to five F_2_ samples and not in the F_1_ parent (a parental somatic mutation should be mosaic in the parent). Only four putative candidates were found in intraspecific (*P. persica*) group, two were found in the interspecific group. This adds very little to the sum tally of new mutations, again suggesting, but not proving, that somatic mutation is not explaining the near doubling seen in the hybrid.

These results argue against somatic mutation as the single cause of the difference between the interspecific and intraspecific crosses. None of these results, however, definitely exclude the possibility. We, thus, conclude that the mutation rate observed in the interspecific class is consistent with increased mutation rates in hybrids, but does not constitute definitive evidence for this effect.

## 4. Discussion

Mutation rates per bp per generation per haploid genome in peach and *Arabidopsis* are similar. Even if we take our upper-bound as being the rate from the interspecific cross (group III), the estimate is about double that from homozygous selfing *Arabidopsis*. While it is expected, from prior indirect substitutional data of woody perennials [6–8], that peach might have a lower per unit time mutation rate than *Arabidopsis*, it is noteworthy that the average base mutation rate in peach, with a generation cycle of at least 3 years, is very similar to the base mutation rate in *Arabidopsis* (7.0 to 7.4 × 10^−9^) [4,54], which requires only 5–6 weeks from seed to seed [55], at least under ideal conditions. Note that one *Arabidopsis* estimate also comes from leaf to leaf single-generation estimation with comparable stringency of calling. Thus, peach appears to have an effective mutation rate approximately an order of magnitude lower than that in *Arabidopsis*, when assayed per unit time, even taking the liberal group III estimation. That we see a lower rate when one parent is 200 years old further underscores this result.

The low apparent rate in peach has several further corollaries. Peach is much larger than *Arabidopsis*, so the absolute number of cell divisions from zygote to zygote is likely to be higher in peach. If so, the apparent lack of difference in mutation rate per generation most probably reflects a difference in the per replication mutation rate, with peach having fewer mutations per cell division. We note that the per cell division rate is possibly higher than might be extrapolated from the between generation rate, as some mutations must be cell lethal and thus not recovered. However, we see little evidence for selection on the observed mutational profile, suggesting that such cell selection is relatively rare.

We find evidence consistent with the hybridization–mutation coupling. We cannot fully exclude somatic mutation as accounting for the difference between group I and group III, although some evidence is suggestive that this is unlikely to explain all of the difference. Nonetheless, we conclude that our evidence is consistent with the hybridization–mutation hypothesis, but with the caveat that somatic mutation may yet explain, part or all of, the difference.

That we find mutations in the vicinity of heterozygous sites is consistent with the possibility of heterozygous-induced mutation. However, it may also be consistent with the heterozygous-associated mutation. In zones of the genome relatively permissive for mutation, we expect to have higher heterozygosity. If the genome level mutation rate increases and if such mutations are more common in the permissive domains, then they will be expected to be closer to heterozygous sites, even if such sites are not mutagenic *per se*. We can, in summary, conclude that we failed to falsify the heterozygous-induced mutation hypothesis, first proposed by Emerson [26], while the experiment could have falsified it had we observed a uniform distribution of new mutations.

## Supplementary Material

Supplementary Figures and Tables

## Supplementary Material

Supplementary Methods

## Data accessibility

The sequence data from this study have been submitted to the NCBI BioProject database (http://www.ncbi.nlm.nih.gov/bioproject/) under accession number SRP071980.
