# Supplementary material for: Mutation rate analysis via parent–progeny sequencing of the perennial peach. I. A low rate in woody perennials and a higher mutagenicity in hybrids
Source: Proc Biol Sci. 2016 Oct 26;283(1841):20161016. doi: 10.1098/rspb.2016.1016 (PMC5095371; doi:10.1098/rspb.2016.1016)
Supplement: Supplementary Figures and Tables [file rspb20161016supp1.docx]

Supplementary Figures and Tables for:

**Mutation rate analysis via parent-progeny sequencing of the perennial peach I. A low rate in woody perennials and a higher mutagenicity in hybrids**

Zhengqing Xie, Long Wang, Lirong Wang, Zhiqiang Wang, Zhenhua Lu, Dacheng Tian, Sihai Yang, Laurence D. Hurst

DOI: 10.1098/rspb.2016.1016

Supplemental Fig S1**.** **Pedigree information of peach F_2_s of the interspecific group (III).** In this pedigree, the 2005-W is the F_1_ offspring of ZXST-1 (*Prunus* *davidiana*) and 96-7-52 (*Prunus persica*). In this pedigree, 30 selfed F_2_s (NE1 to 30) from a F_1_ tree of 2005-W were employed to detect *de novo* mutations. In addition, except for 91-1-5 and 96-7-52 which have already died, the other four parents (HR-E, MLWL-E, SG-E and ZXST-1 have been sequenced in this study. HR-E (Sunred), *Prunus persica* (L.) Batsch; MLWL-E (Maravilha), *Prunus persica* (L.) Batsch. SG-E (Shu Guang) *Prunus persica* (L.) Batsch; ZXST-1, (Zhou Xing Shan Tao), *Prunus* *davidiana*(Carr.)　Franch.


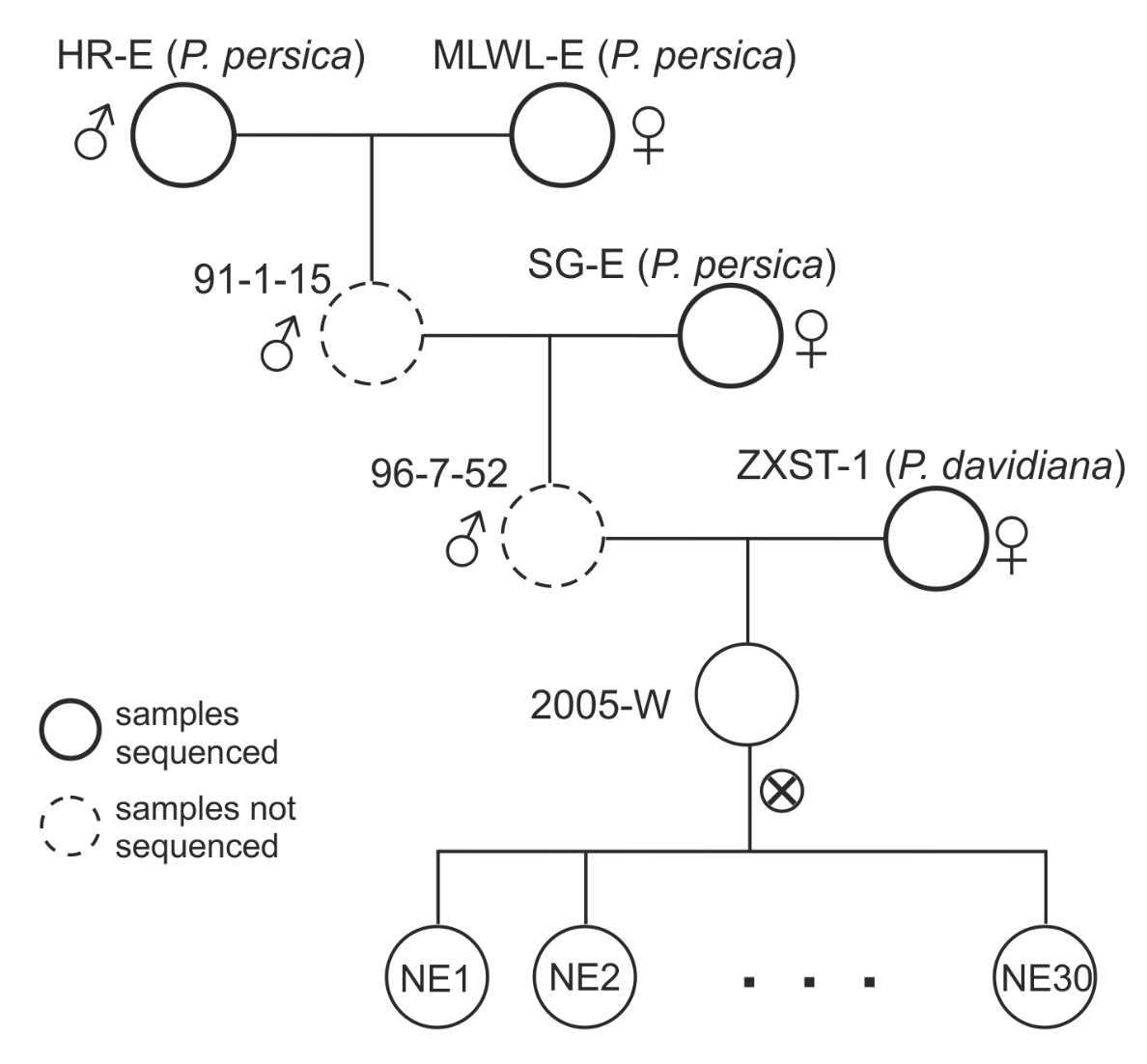


Supplemental Fig S2**.** **Sampling timeline of intraspecific group I and interspecific group III.** All trees were sampled in 2015. Leaves were collected from an arbitrary branch of a tree or from the whole saplings.

**
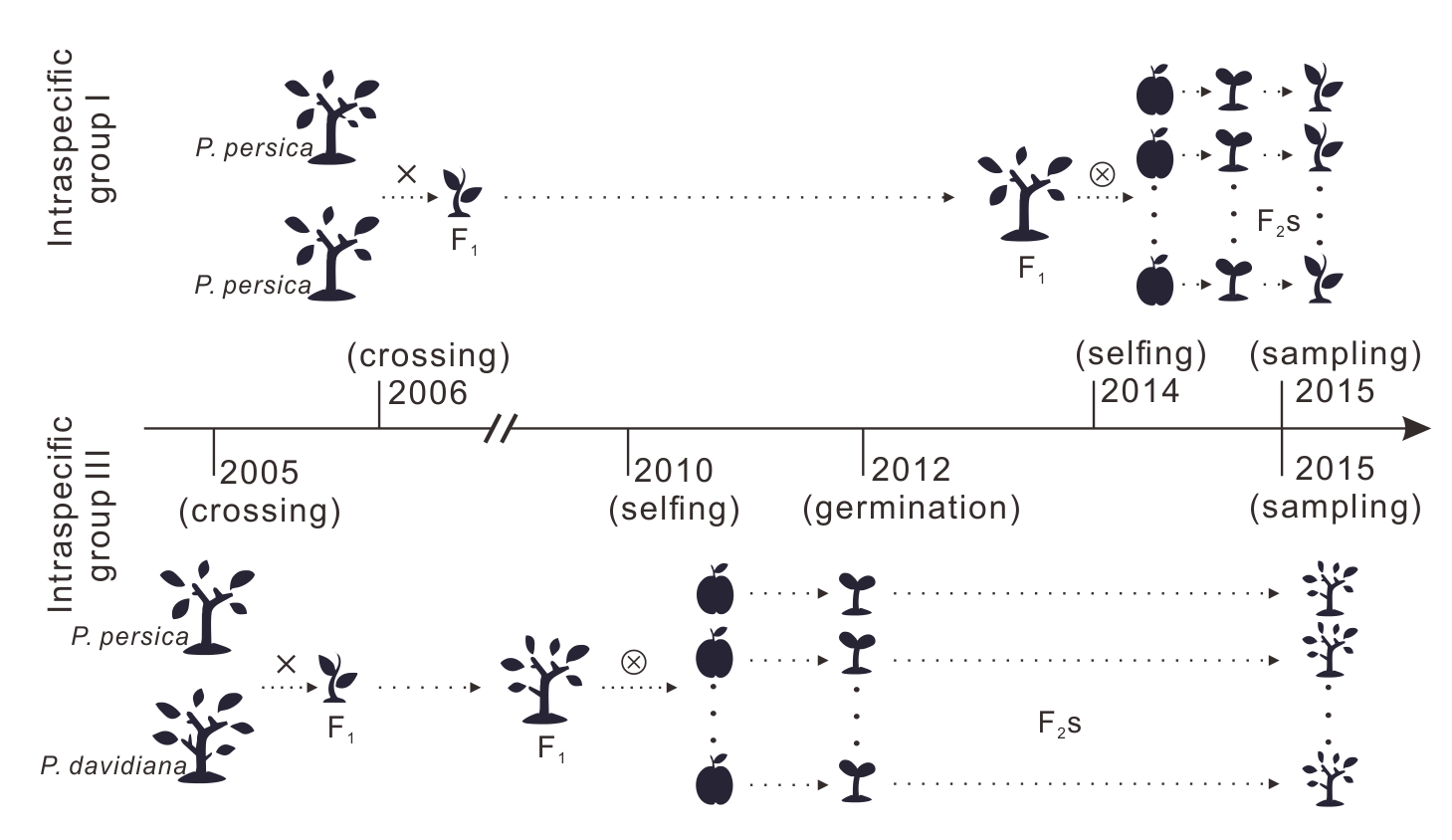
**

Supplemental Fig S3**. Identified mutations in different compartments of (A) intraspecific (*P. persica*) F_2_ samples and (B) interspecific F_2_ samples.** The percentage in brackets reflect the proportion of the genome that is heterozygous (Het.) or homozygous (Hom.).

**
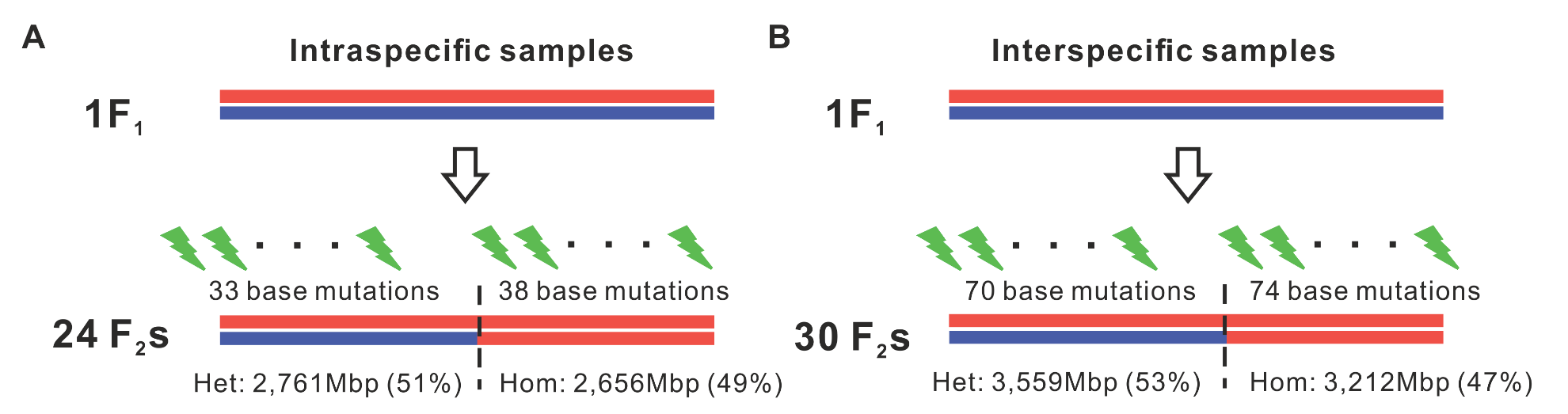
**

**Supplemental** **Table S1. Sequencing depth and genome coverage (%) of all samples.**

| **Type** | **Sample** | **Clean data (Gb)** | **Sequencing depth** | **Uniquely mapped depth*** | **Uniquely mapped coverage (≥5 reads)** |
| --- | --- | --- | --- | --- | --- |
| **Intraspecific groups** | | | | | |
| *P. persica* F_1_ sample | 144F1-3 | 11.0 | 48.4 | 31.7 | 93.3 |
| *P. persica* F_2_ samples | 144F2-1 | 10.6 | 46.6 | 29.9 | 90.8 |
|  | 144F2-2 | 12.5 | 54.9 | 35.0 | 92.8 |
|  | 144F2-3 | 11.1 | 48.7 | 31.0 | 91.1 |
|  | 144F2-4 | 11.6 | 51.2 | 32.9 | 92.2 |
|  | 144F2-5 | 11.7 | 51.4 | 33.9 | 91.9 |
|  | 144F2-6 | 11.7 | 51.6 | 33.0 | 92.3 |
|  | 144F2-7 | 11.8 | 51.8 | 33.7 | 92.8 |
|  | 144F2-8 | 10.1 | 44.5 | 28.7 | 91.3 |
|  | 144F2-9 | 10.3 | 45.4 | 28.1 | 91 |
|  | 144F2-10 | 12.3 | 54.0 | 33.8 | 92.9 |
|  | 144F2-11 | 12.2 | 53.8 | 33.2 | 92 |
|  | 144F2-12 | 14.5 | 63.6 | 38.1 | 90.9 |
|  | 144F2-13 | 10.8 | 47.3 | 30.8 | 92.5 |
|  | 144F2-14 | 10.4 | 45.9 | 28.2 | 91.4 |
|  | 144F2-15 | 11.8 | 51.9 | 32.1 | 91.8 |
|  | 144F2-16 | 10.2 | 45.0 | 27.0 | 91.8 |
|  | 144F2-17 | 10.7 | 46.8 | 29.7 | 92.2 |
|  | 144F2-18 | 12.3 | 54.0 | 32.2 | 92.5 |
|  | 144F2-19 | 12.8 | 56.4 | 32.2 | 91.7 |
|  | 144F2-20 | 12.1 | 53.1 | 31.9 | 92.6 |
|  | 144F2-21 | 11.5 | 50.6 | 30.7 | 92.8 |
|  | 144F2-22 | 11.0 | 48.2 | 28.4 | 91.7 |
|  | 144F2-23 | 11.2 | 49.4 | 30.5 | 92.3 |
|  | 144F2-24 | 11.0 | 48.4 | 30.0 | 92.8 |
| *P. mira* F_1_ sample | GZ-1 | 10.67 | 46.9 | 26.6 | 78 |
| *P. mira*  F_2_ samples | GZTH-5 | 11.32 | 49.8 | 25.3 | 77.4 |
|  | GZTH-8** | 11.94 | 52.5 | 14.3 | 74.9 |
|  | GZTH-S1 | 10.04 | 44.1 | 26.8 | 76.6 |
|  | GZTH-S2 | 8.72 | 38.3 | 23.3 | 76.7 |
|  | GZTH-S3 | 11.78 | 51.8 | 30.1 | 77.4 |
|  | GZTH-S4 | 12.07 | 53.1 | 31.6 | 77.3 |
|  | GZTH-S5 | 12.16 | 53.5 | 21.7 | 76.8 |
|  | GZTH-S7 | 10.48 | 46.1 | 27.5 | 77.2 |
|  | GZTH-S8 | 11.94 | 52.5 | 31.6 | 77.9 |
|  | GZTH-S9 | 10.83 | 47.6 | 28.6 | 77.6 |
| **Interspecific group (*P. persica* × *P. davidiana*)** | | | | | |
| Parent Samples | HR-E | 10.9 | 47.8 | 35.3 | 92.7 |
|  | MLWL-E | 10.5 | 46.2 | 32.9 | 93.2 |
|  | SG-E | 12.2 | 53.8 | 38.6 | 94.7 |
|  | ZXST-1 | 12.5 | 54.8 | 32.8 | 79.5 |
| Heterozygous F_1_ sample | 2005-W | 11.6 | 50.9 | 33.3 | 92.4 |
| Heterozygous F_2_ samples | NE1 | 13.5 | 59.2 | 37.0 | 89.1 |
|  | NE2 | 11.1 | 48.9 | 32.8 | 91.8 |
|  | NE3 | 12.3 | 53.9 | 34.9 | 88.8 |
|  | NE4 | 12.5 | 55.1 | 32.4 | 87 |
|  | NE5 | 11.5 | 50.4 | 32.2 | 86 |
|  | NE6 | 12.0 | 52.9 | 32.1 | 87.6 |
|  | NE7 | 11.7 | 51.5 | 33.5 | 90.2 |
|  | NE8 | 11.4 | 50.3 | 31.4 | 91.3 |
|  | NE9 | 11.5 | 50.6 | 33.3 | 89.5 |
|  | NE10 | 12.1 | 53.1 | 34.6 | 91.8 |
|  | NE11 | 12.2 | 53.8 | 34.8 | 90.7 |
|  | NE12 | 12.0 | 52.6 | 35.1 | 89.6 |
|  | NE13 | 11.2 | 49.2 | 32.2 | 90.2 |
|  | NE14 | 9.9 | 43.6 | 28.9 | 87.6 |
|  | NE15 | 12.7 | 56.0 | 34.6 | 90.3 |
|  | NE16 | 11.9 | 52.4 | 33.7 | 87.6 |
|  | NE17 | 11.9 | 52.4 | 32.4 | 87 |
|  | NE18 | 13.6 | 59.8 | 38.8 | 92.6 |
|  | NE19 | 12.5 | 55.1 | 32.8 | 85 |
|  | NE20 | 12.1 | 53.3 | 33.9 | 88.5 |
|  | NE21 | 11.8 | 52.0 | 32.0 | 89 |
|  | NE22 | 15.0 | 65.8 | 38.7 | 89.8 |
|  | NE23 | 14.3 | 62.7 | 37.9 | 88.7 |
|  | NE24 | 13.7 | 60.3 | 36.6 | 86.4 |
|  | NE25 | 13.7 | 60.3 | 37.5 | 89.9 |
|  | NE26 | 11.2 | 49.5 | 30.7 | 88.7 |
|  | NE27 | 10.4 | 45.6 | 30.2 | 87.7 |
|  | NE28 | 12.5 | 55.1 | 35.6 | 90.2 |
|  | NE29 | 11.2 | 49.3 | 33.3 | 90.4 |
|  | NE30 | 11.1 | 48.7 | 30.9 | 87.6 |

*Reads with a mapping quality ≥20 were considered as uniquely mapped;

**Sample poorly sequenced and only used in comparing to exclude false positives.

**Supplemental** **Table S2. Fractions of callable simulated mutated sites.**

| Groups | Valid Sites* | Uncallable Sites | | | | | Callable Sites | | Fractions of Callable Sites (%) |
| --- | --- | --- | --- | --- | --- | --- | --- | --- | --- |
|  |  | Not Called by any Callers | Low Depth** (<5) in Focal Sample | Strand Bias in Focal Sample | No Depth in Parent Samples | No Depth in ≥ 6 other F2s | Recovered as substitutions | Not Recovered |  |
| Intra group I | 995 | 97 | 39 | 13 | 0 | 7 | 837 | 2 | 84.3 |
| Intra group II | 992 | 265 | 56 | 27 | 0 | 0 | 638 | 6 | 64.9 |
| Inter group III | 991 | 110 | 24 | 13 | 71 | 11 | 755 | 7 | 76.9 |

*Site happened to be a pre-existing variant locus which has an identical allele with the synthesized mutation allele was considered as invalid; **Depth refers to number of covered reads with mapping quality ≥ 20.

**Supplemental** **Table S3. List of the *de novo* mutations.**

| **No.** | **Mutation Position** | **Mutation Type** | **Sample** | **Regions** |
| --- | --- | --- | --- | --- |
| **Intraspecific group I (*P. persica*)** | | | | |
| 1 | Pp01:2415880 | SNP:C→T | 144F2-13 | intergenic |
| 2 | Pp01:3304880 | SNP:G→A | 144F2-10 | intergenic |
| 3 | Pp01:3898323 | SNP:C→T | 144F2-2 | intergenic |
| 4 | Pp01:9110065 | SNP:G→A | 144F2-8 | intergenic |
| 5 | Pp01:11971314 | SNP:G→A | 144F2-5 | UTR |
| 6 | Pp01:14552283 | SNP:G→A | 144F2-24 | intergenic |
| 7 | Pp01:15022348 | SNP:G→A | 144F2-13 | intergenic |
| 8 | Pp01:17835051 | SNP:T→A | 144F2-17 | intergenic |
| 9 | Pp01:17905203 | SNP:A→G | 144F2-6 | intergenic |
| 10 | Pp01:21413721 | SNP:G→A | 144F2-3 | intergenic |
| 11 | Pp01:23614684 | SNP:A→G | 144F2-3 | intergenic |
| 12 | Pp01:26803687 | SNP:C→T | 144F2-12 | intergenic |
| 13 | Pp01:27352008 | SNP:T→G | 144F2-4 | intergenic |
| 14 | Pp01:28743588 | SNP:C→T | 144F2-14 | intergenic |
| 15 | Pp01:29048923 | SNP:T→A | 144F2-12 | intergenic |
| 16 | Pp01:30225059 | SNP:G→A | 144F2-15 | intergenic |
| 17 | Pp01:31348115-31348116 | Ins:AT | 144F2-18 | intergenic |
| 18 | Pp01:33642546 | SNP:C→T | 144F2-21 | non-synonymous |
| 19 | Pp01:34641431-34641436 | Del:CTTTTT | 144F2-18 | intergenic |
| 20 | Pp01:35285918 | SNP:C→T | 144F2-17 | non-synonymous |
| 21 | Pp01:37814273-37814274 | Ins:A | 144F2-23 | intron |
| 22 | Pp01:41029336 | SNP:G→A | 144F2-15 | synonymous |
| 23 | Pp01:41565860-41565877 | Del:AAATGGTACAATTTGAGC | 144F2-19 | UTR |
| 24 | Pp01:46423173 | SNP:G→T | 144F2-21 | intergenic |
| 25 | Pp01:47406109-47406109 | Del:A | 144F2-20 | intergenic |
| 26 | Pp02:526006 | SNP:G→A | 144F2-1 | intergenic |
| 27 | Pp02:2620047-2620048 | Ins:A | 144F2-10 | intergenic |
| 28 | Pp02:6320036 | SNP:G→A | 144F2-16 | intergenic |
| 29 | Pp02:6896792 | SNP:T→A | 144F2-13 | intergenic |
| 30 | Pp02:8110096 | SNP:A→G | 144F2-19 | intergenic |
| 31 | Pp02:10020826 | SNP:G→A | 144F2-16 | intron |
| 32 | Pp02:10512064 | SNP:T→C | 144F2-23 | intergenic |
| 33 | Pp02:24686938-24686939 | Ins:CT | 144F2-15 | intron |
| 34 | Pp02:26806561 | SNP:G→A | 144F2-21 | intron |
| 35 | Pp02:28018017 | SNP:G→A | 144F2-6 | intergenic |
| 36 | Pp02:28793664-28793665 | Del:AC | 144F2-8 | intron |
| 37 | Pp03:1013972 | SNP:G→A | 144F2-19 | non-synonymous |
| 38 | Pp03:2664016-2664017 | Ins:A | 144F2-24 | UTR |
| 39 | Pp03:4594851 | SNP:C→A | 144F2-5 | intergenic |
| 40 | Pp03:7538752 | SNP:C→A | 144F2-6 | intergenic |
| 41 | Pp03:10145439 | SNP:T→C | 144F2-19 | UTR |
| 42 | Pp03:10197738 | SNP:C→T | 144F2-5 | intergenic |
| 43 | Pp03:11916515 | SNP:T→C | 144F2-1 | UTR |
| 44 | Pp03:13046463 | SNP:G→A | 144F2-5 | intergenic |
| 45 | Pp03:15157990 | SNP:C→T | 144F2-22 | intergenic |
| 46 | Pp04:5327760 | SNP:C→T | 144F2-8 | intergenic |
| 47 | Pp04:5922624 | SNP:C→T | 144F2-20 | intergenic |
| 48 | Pp04:7647744 | SNP:G→A | 144F2-23 | non-synonymous |
| 49 | Pp04:9583771 | SNP:T→A | 144F2-8 | intergenic |
| 50 | Pp04:17620273-17620273 | Del:T | 144F2-6 | intergenic |
| 51 | Pp04:21397354 | SNP:C→T | 144F2-13 | intergenic |
| 52 | Pp04:23458873 | SNP:G→A | 144F2-17 | intergenic |
| 53 | Pp04:24836792 | SNP:G→T | 144F2-18 | intergenic |
| 54 | Pp05:71042 | SNP:G→A | 144F2-8 | intergenic |
| 55 | Pp05:2940952 | SNP:G→A | 144F2-18 | intergenic |
| 56 | Pp05:6858820 | SNP:G→A | 144F2-23 | intergenic |
| 57 | Pp05:8853207-8853207 | Del:T | 144F2-19 | frameshift |
| 58 | Pp05:12340628 | SNP:G→A | 144F2-8 | intergenic |
| 59 | Pp05:18484080 | SNP:C→T | 144F2-2 | intergenic |
| 60 | Pp06:2115689 | SNP:A→G | 144F2-20 | non-synonymous |
| 61 | Pp06:2965791 | SNP:A→T | 144F2-24 | intergenic |
| 62 | Pp06:9060517 | SNP:C→T | 144F2-24 | intron |
| 63 | Pp06:12477019 | SNP:G→A | 144F2-9 | intergenic |
| 64 | Pp06:15925523 | SNP:G→A | 144F2-16 | intergenic |
| 65 | Pp06:16051821 | SNP:T→C | 144F2-16 | intergenic |
| 66 | Pp06:20873925 | SNP:G→A | 144F2-9 | intergenic |
| 67 | Pp06:23787438 | SNP:G→A | 144F2-21 | intergenic |
| 68 | Pp07:248813 | SNP:T→G | 144F2-1 | intergenic |
| 69 | Pp07:647915 | SNP:G→A | 144F2-14 | intergenic |
| 70 | Pp07:1886889 | SNP:A→G | 144F2-9 | intergenic |
| 71 | Pp07:4083616 | SNP:C→T | 144F2-10 | intron |
| 72 | Pp07:4882253 | SNP:C→A | 144F2-6 | intergenic |
| 73 | Pp07:6512245 | SNP:T→G | 144F2-17 | non-synonymous |
| 74 | Pp07:10660844 | SNP:G→A | 144F2-17 | intergenic |
| 75 | Pp07:10767301 | SNP:G→A | 144F2-14 | intergenic |
| 76 | Pp07:11373380 | SNP:A→T | 144F2-18 | intergenic |
| 77 | Pp07:14278052 | SNP:C→T | 144F2-18 | intergenic |
| 78 | Pp08:1627100 | SNP:C→T | 144F2-21 | intergenic |
| 79 | Pp08:5913378 | SNP:G→A | 144F2-5 | intergenic |
| 80 | Pp08:6026880 | SNP:T→C | 144F2-15 | intergenic |
| 81 | Pp08:18685443 | SNP:A→T | 144F2-19 | intergenic |
| 82 | Pp08:21774245 | SNP:C→A | 144F2-8 | intergenic |
| **Intraspecific group II (*P. mira*)** | | | | |
| 1 | Pp01:28998848 | SNP:A→G | GZTH-S4 | intergenic |
| 2 | Pp01:32494000 | SNP:T→A | GZTH-S8 | intergenic |
| 3 | Pp01:33384324 | SNP:G→A | GZTH-S4 | intergenic |
| 4 | Pp01:43001858 | SNP:A→T | GZTH-S4 | intergenic |
| 5 | Pp01:44098708 | SNP:C→T | GZTH-S8 | UTR |
| 6 | Pp02:1795391 | SNP:A→T | GZTH-S5 | intergenic |
| 7 | Pp02:10849640 | SNP:C→T | GZTH-S5 | intergenic |
| 8 | Pp02:15979477 | SNP:C→A | GZTH-S3 | intergenic |
| 9 | Pp02:18791853 | SNP:G→A | GZTH-5 | intergenic |
| 10 | Pp02:25863307-25863308 | Ins:TA | GZTH-5 | intergenic |
| 11 | Pp02:30404719 | SNP:G→T | GZTH-S3 | intergenic |
| 12 | Pp03:11562621-11562628 | Del:AAATAAAT | GZTH-S8 | intron |
| 13 | Pp03:12745271 | SNP:T→A | GZTH-S4 | intergenic |
| 14 | Pp03:23396936-23396937 | Ins:CCG | GZTH-S1 | intergenic |
| 15 | Pp04:12828006-12828016 | Del:TGTTTTGGTGG | GZTH-S5 | intron |
| 16 | Pp05:4464637 | SNP:T→G | GZTH-5 | intergenic |
| 17 | Pp05:9418975 | SNP:G→T | GZTH-S2 | UTR |
| 18 | Pp05:9882528 | SNP:G→A | GZTH-S1 | UTR |
| 19 | Pp06:1087492 | SNP:C→A | GZTH-5 | intergenic |
| 20 | Pp06:1423206-1423207 | Ins:A | GZTH-5 | intergenic |
| 21 | Pp06:6819139 | SNP:G→A | GZTH-S9 | intergenic |
| 22 | Pp06:9295196 | SNP:C→G | GZTH-S1 | intron |
| 23 | Pp06:13704004 | SNP:A→G | GZTH-S9 | intergenic |
| 24 | Pp06:23049769-23049770 | Ins:AT | GZTH-5 | intergenic |
| 25 | Pp06:24082403 | SNP:G→A | GZTH-S3 | intergenic |
| 26 | Pp06:24836312 | SNP:C→A | GZTH-S5 | UTR |
| 27 | Pp06:27321334 | SNP:A→G | GZTH-S7 | intron |
| 28 | Pp06:30435744-30435744 | Del:A | GZTH-5 | intergenic |
| 29 | Pp07:2520073 | SNP:C→T | GZTH-5 | intergenic |
| 30 | Pp07:18575105 | SNP:T→A | GZTH-S4 | non-synonymous |
| 31 | Pp08:5821817 | SNP:A→C | GZTH-S5 | intergenic |
| 32 | Pp08:9836047 | SNP:T→C | GZTH-S3 | intergenic |
| **Interspecific group III** | | | | |
| 1 | Pp01:265281 | SNP:C→A | NE23 | intergenic |
| 2 | Pp01:1932670-1932671 | Ins:AT | NE15 | intergenic |
| 3 | Pp01:2830461 | SNP:A→G | NE23 | intron |
| 4 | Pp01:3595346-3595347 | Ins:AA | NE28 | UTR |
| 5 | Pp01:4516524 | SNP:C→T | NE19 | intron |
| 6 | Pp01:4758436 | SNP:T→C | NE2 | intergenic |
| 7 | Pp01:5230269 | SNP:G→A | NE25 | UTR |
| 8 | Pp01:7725462 | SNP:A→C | NE10 | intergenic |
| 9 | Pp01:8092829 | SNP:C→T | NE21 | intergenic |
| 10 | Pp01:8351563 | SNP:G→A | NE12 | intergenic |
| 11 | Pp01:8567743 | SNP:A→T | NE9 | intron |
| 12 | Pp01:8679738 | SNP:T→G | NE28 | intergenic |
| 13 | Pp01:9518708 | SNP:T→C | NE23 | intergenic |
| 14 | Pp01:10339774 | SNP:G→A | NE16 | intergenic |
| 15 | Pp01:10771510 | SNP:C→T | NE29 | intergenic |
| 16 | Pp01:10832701 | SNP:G→A | NE3 | intergenic |
| 17 | Pp01:11156638 | SNP:G→A | NE2 | intergenic |
| 18 | Pp01:11430535 | SNP:G→A | NE17 | non-synonymous |
| 19 | Pp01:14760505 | SNP:C→T | NE5 | intergenic |
| 20 | Pp01:19305326 | SNP:G→T | NE20 | intergenic |
| 21 | Pp01:20872145 | SNP:T→A | NE20 | intergenic |
| 22 | Pp01:21661383 | SNP:A→T | NE26 | intergenic |
| 23 | Pp01:24218128 | SNP:G→A | NE7 | intergenic |
| 24 | Pp01:24801633 | SNP:G→C | NE1 | synonymous |
| 25 | Pp01:26107662 | SNP:C→T | NE15 | intergenic |
| 26 | Pp01:26569254 | SNP:G→T | NE8 | UTR |
| 27 | Pp01:27020393 | SNP:G→T | NE2 | non-synonymous |
| 28 | Pp01:27939835 | SNP:G→T | NE28 | intergenic |
| 29 | Pp01:29764628-29764653 | Del:CTCTTCGCTTCACACGCGAGGTGAGTT | NE2 | intergenic |
| 30 | Pp01:33237674 | SNP:A→T | NE15 | intergenic |
| 31 | Pp01:33298890 | SNP:C→T | NE1 | intergenic |
| 32 | Pp01:33375562 | SNP:G→A | NE5 | intergenic |
| 33 | Pp01:35251770 | SNP:G→A | NE10 | intergenic |
| 34 | Pp01:35325329-35325330 | Ins:C | NE10 | frameshift |
| 35 | Pp01:35905588 | SNP:C→A | NE20 | intergenic |
| 36 | Pp01:36560657 | SNP:T→A | NE11 | intergenic |
| 37 | Pp01:36794004 | SNP:C→T | NE19 | intergenic |
| 38 | Pp01:39919567 | SNP:G→A | NE8 | intergenic |
| 39 | Pp01:40595481-40595542 | Del:GCGCCAAAAGTGAGGGGTAAGAGTGTAAATAGTCACTAGACTCAACTCCCTCCTCTAGATCCTTTTTTCTT | NE12 | intergenic |
| 40 | Pp01:41155606 | SNP:G→A | NE9 | intergenic |
| 41 | Pp01:41877504 | SNP:G→A | NE13 | intergenic |
| 42 | Pp01:42159985 | SNP:C→T | NE15 | UTR |
| 43 | Pp01:42623620 | SNP:G→A | NE17 | intergenic |
| 44 | Pp01:45595227 | SNP:G→A | NE13 | intergenic |
| 45 | Pp01:47328342 | SNP:A→G | NE11 | non-synonymous |
| 46 | Pp01:47525239 | SNP:C→T | NE4 | UTR |
| 47 | Pp02:3459078-3459078 | Del:T | NE2 | intron |
| 48 | Pp02:4477666-4477667 | Ins:CAAC | NE30 | intron |
| 49 | Pp02:4878900 | SNP:C→T | NE4 | intron |
| 50 | Pp02:5208765 | SNP:C→T | NE4 | intergenic |
| 51 | Pp02:8539763 | SNP:G→A | NE10 | intergenic |
| 52 | Pp02:9184140 | SNP:C→T | NE30 | intergenic |
| 53 | Pp02:9224015 | SNP:G→A | NE13 | intergenic |
| 54 | Pp02:9309414 | SNP:T→G | NE21 | intergenic |
| 55 | Pp02:9331116 | SNP:A→G | NE4 | UTR |
| 56 | Pp02:9918537 | SNP:G→A | NE18 | intergenic |
| 57 | Pp02:10681566 | SNP:T→C | NE26 | intergenic |
| 58 | Pp02:10889574-10889576 | Del:TCA | NE20 | intergenic |
| 59 | Pp02:12189711 | SNP:T→C | NE20 | intergenic |
| 60 | Pp02:12237928-12237929 | Del:TT | NE21 | intron |
| 61 | Pp02:12868614-12868614 | Del:A | NE21 | intergenic |
| 62 | Pp02:15122104 | SNP:T→C | NE19 | intergenic |
| 63 | Pp02:16053475-16053476 | Ins:AT | NE10 | intergenic |
| 64 | Pp02:22450221 | SNP:C→G | NE11 | UTR |
| 65 | Pp02:23740151 | SNP:G→A | NE26 | non-synonymous |
| 66 | Pp02:24302480 | SNP:T→A | NE20 | intergenic |
| 67 | Pp02:24530252-24530253 | Ins:T | NE27 | frameshift |
| 68 | Pp02:26795822 | SNP:T→C | NE9 | intron |
| 69 | Pp03:2133825 | SNP:G→T | NE21 | intron |
| 70 | Pp03:2412845 | SNP:C→T | NE10 | intergenic |
| 71 | Pp03:3343562 | SNP:T→G | NE21 | intron |
| 72 | Pp03:4050927 | SNP:C→T | NE12 | non-synonymous |
| 73 | Pp03:6323961 | SNP:C→T | NE12 | intergenic |
| 74 | Pp03:7528958 | SNP:C→T | NE9 | intron |
| 75 | Pp03:13141098 | SNP:G→A | NE23 | intergenic |
| 76 | Pp03:14438606 | SNP:C→T | NE20 | intergenic |
| 77 | Pp03:16809829 | SNP:G→T | NE20 | intergenic |
| 78 | Pp03:17588590 | SNP:T→G | NE28 | non-synonymous |
| 79 | Pp03:17723369 | SNP:C→T | NE13 | intergenic |
| 80 | Pp03:17745760 | SNP:A→T | NE21 | intergenic |
| 81 | Pp03:22352568-22352574 | Del:AGAAGAC | NE13 | UTR |
| 82 | Pp03:23834241-23834242 | Ins:T | NE27 | frameshift |
| 83 | Pp03:25357063 | SNP:T→A | NE30 | UTR |
| 84 | Pp03:25622439 | SNP:C→T | NE4 | UTR |
| 85 | Pp04:3117461 | SNP:G→A | NE4 | UTR |
| 86 | Pp04:7049585 | SNP:G→A | NE12 | intergenic |
| 87 | Pp04:8135043 | SNP:G→A | NE2 | intergenic |
| 88 | Pp04:8828966-8828967 | Ins:T | NE19 | intron |
| 89 | Pp04:11858076 | SNP:T→C | NE24 | synonymous |
| 90 | Pp04:12499748 | SNP:G→A | NE19 | UTR |
| 91 | Pp04:16641286 | SNP:C→T | NE7 | intergenic |
| 92 | Pp04:18021984 | SNP:C→T | NE4 | intergenic |
| 93 | Pp04:20733890 | SNP:C→T | NE2 | intergenic |
| 94 | Pp04:20881466 | SNP:G→A | NE18 | intergenic |
| 95 | Pp04:20986834 | SNP:T→C | NE22 | intergenic |
| 96 | Pp04:22231542 | SNP:G→T | NE5 | intergenic |
| 97 | Pp04:22257387 | SNP:C→T | NE29 | intergenic |
| 98 | Pp04:22365129 | SNP:C→T | NE6 | intergenic |
| 99 | Pp04:23408105 | SNP:C→A | NE18 | intergenic |
| 100 | Pp04:23439159 | SNP:C→T | NE19 | intron |
| 101 | Pp05:166216 | SNP:G→A | NE1 | intergenic |
| 102 | Pp05:1447170-1447171 | Ins:AGAGGG | NE5 | inframe |
| 103 | Pp05:2899257 | SNP:C→T | NE27 | intergenic |
| 104 | Pp05:9320533 | SNP:C→T | NE24 | synonymous |
| 105 | Pp05:10163013 | SNP:G→T | NE4 | non-synonymous |
| 106 | Pp05:12611918 | SNP:C→T | NE12 | intergenic |
| 107 | Pp05:13733774 | SNP:G→A | NE21 | intergenic |
| 108 | Pp05:13734230 | SNP:C→T | NE21 | non-synonymous |
| 109 | Pp05:13838996 | SNP:C→T | NE6 | synonymous |
| 110 | Pp05:14684185 | SNP:A→G | NE21 | UTR |
| 111 | Pp05:16746372 | SNP:T→C | NE10 | intergenic |
| 112 | Pp05:17097771 | SNP:G→A | NE8 | intergenic |
| 113 | Pp06:747769 | SNP:G→A | NE5 | UTR |
| 114 | Pp06:856715 | SNP:C→A | NE15 | intergenic |
| 115 | Pp06:2845265 | SNP:T→G | NE12 | UTR |
| 116 | Pp06:3408495 | SNP:C→G | NE17 | intergenic |
| 117 | Pp06:5181026 | SNP:C→T | NE18 | intergenic |
| 118 | Pp06:6866166 | SNP:C→T | NE26 | intergenic |
| 119 | Pp06:7237513 | SNP:C→T | NE10 | intergenic |
| 120 | Pp06:7690900 | SNP:C→T | NE7 | intergenic |
| 121 | Pp06:8325904-8325905 | Ins:A | NE3 | intergenic |
| 122 | Pp06:9854413-9854413 | Del:A | NE17 | intron |
| 123 | Pp06:11031043 | SNP:G→A | NE2 | intergenic |
| 124 | Pp06:11181374-11181374 | Del:AT | NE11 | intergenic |
| 125 | Pp06:15265633 | SNP:G→T | NE22 | intergenic |
| 126 | Pp06:15710800 | SNP:C→T | NE29 | intergenic |
| 127 | Pp06:16177872 | SNP:T→C | NE9 | intergenic |
| 128 | Pp06:16339861 | SNP:G→A | NE25 | intergenic |
| 129 | Pp06:18206068-18206069 | Ins:T | NE9 | intron |
| 130 | Pp06:18235948-18235949 | Ins:GCTGGTGA | NE4 | intergenic |
| 131 | Pp06:18659811-18659812 | Ins:AGAGG | NE12 | intergenic |
| 132 | Pp06:18866240 | SNP:C→T | NE21 | intergenic |
| 133 | Pp06:19221556 | SNP:G→A | NE27 | intergenic |
| 134 | Pp06:19502904-19502905 | Ins:A | NE19 | frameshift |
| 135 | Pp06:20164716 | SNP:G→A | NE21 | intergenic |
| 136 | Pp06:22423518 | SNP:C→T | NE3 | intergenic |
| 137 | Pp06:22466995 | SNP:A→T | NE23 | intron |
| 138 | Pp06:23703306 | SNP:C→T | NE7 | intergenic |
| 139 | Pp06:24417673 | SNP:C→T | NE28 | intergenic |
| 140 | Pp06:25153327 | SNP:G→T | NE1 | non-synonymous |
| 141 | Pp06:27229563-27229564 | Ins:GAGA | NE16 | intron |
| 142 | Pp06:27724945 | SNP:C→T | NE30 | intergenic |
| 143 | Pp06:28325243 | SNP:C→A | NE13 | UTR |
| 144 | Pp06:29462454 | SNP:C→A | NE8 | intergenic |
| 145 | Pp07:960319 | SNP:A→T | NE2 | intron |
| 146 | Pp07:4018394 | SNP:C→T | NE16 | intergenic |
| 147 | Pp07:4123998 | SNP:T→C | NE2 | intergenic |
| 148 | Pp07:6522381 | SNP:A→T | NE25 | non-synonymous |
| 149 | Pp07:6580567 | SNP:C→T | NE26 | non-synonymous |
| 150 | Pp07:7460980 | SNP:T→C | NE6 | intergenic |
| 151 | Pp07:7876219-7876220 | Ins:ATA | NE6 | intron |
| 152 | Pp07:9007741 | SNP:C→A | NE1 | intron |
| 153 | Pp07:9322436 | SNP:C→T | NE4 | intergenic |
| 154 | Pp07:14349006 | SNP:G→A | NE11 | intergenic |
| 155 | Pp07:15242482 | SNP:G→A | NE4 | intergenic |
| 156 | Pp07:18103256 | SNP:C→T | NE25 | UTR |
| 157 | Pp07:19741855 | SNP:G→A | NE22 | intergenic |
| 158 | Pp07:20919858 | SNP:C→T | NE29 | intergenic |
| 159 | Pp08:701436-701437 | Ins:A | NE7 | intergenic |
| 160 | Pp08:1009628 | SNP:C→T | NE4 | intergenic |
| 161 | Pp08:2063718 | SNP:G→T | NE24 | intron |
| 162 | Pp08:2709020 | SNP:G→A | NE4 | intergenic |
| 163 | Pp08:3383643 | SNP:C→T | NE21 | intergenic |
| 164 | Pp08:3690949 | SNP:C→T | NE27 | intergenic |
| 165 | Pp08:4020760 | SNP:T→G | NE9 | intergenic |
| 166 | Pp08:5210115 | SNP:C→T | NE3 | intergenic |
| 167 | Pp08:15440821 | SNP:T→G | NE19 | non-synonymous |
| 168 | Pp08:15440822 | SNP:C→A | NE19 | non-synonymous |
| 169 | Pp08:17742330-17742345 | Del:AGAGAGAGAGAGAGAG | NE28 | intergenic |
| 170 | Pp08:18697941 | SNP:C→G | NE26 | non-synonymous |
| 171 | Pp08:18824389 | SNP:C→T | NE12 | intergenic |
| 172 | Pp08:22365761-22365762 | Ins:TAT | NE6 | intron |

**Supplemental** **Table S4. Nucleotide context of base substitution mutations.**

| **Sequence Context** | | **Base substitutions** | **Genome** | **Ratio (×10^-6^)** |
| --- | --- | --- | --- | --- |
| **Dinucleotides** | CG | 48 | 4,513,022 | 10.64 |
|  | GG | 40 | 9,188,192 | 4.35 |
|  | CC | 38 | 9,197,252 | 4.13 |
|  | GC | 25 | 7,843,093 | 3.19 |
|  | TC | 38 | 13,442,089 | 2.83 |
|  | GT | 32 | 11,356,429 | 2.82 |
|  | CT | 33 | 13,029,109 | 2.53 |
|  | CA | 35 | 15,123,940 | 2.31 |
|  | AC | 26 | 11,380,905 | 2.28 |
|  | GA | 29 | 13,426,172 | 2.16 |
|  | TG | 27 | 15,110,153 | 1.79 |
|  | AG | 23 | 13,002,497 | 1.77 |
|  | TT | 32 | 25,122,693 | 1.27 |
|  | AT | 21 | 20,245,614 | 1.04 |
|  | TA | 15 | 16,079,083 | 0.93 |
|  | AA | 18 | 25,077,566 | 0.72 |
| **Triplets** | CGG | 22 | 964,212 | 22.82 |
|  | GCG | 11 | 764,410 | 14.39 |
|  | ACG | 16 | 1,327,023 | 12.06 |
|  | TCG | 16 | 1,457,340 | 10.98 |
|  | CGT | 14 | 1,320,042 | 10.61 |
|  | CGC | 8 | 768,656 | 10.41 |
|  | CCG | 10 | 964,172 | 10.37 |
|  | CGA | 14 | 1,460,048 | 9.59 |
|  | GAC | 16 | 2,068,280 | 7.74 |
|  | CAG | 19 | 2,493,785 | 7.62 |
|  | GGC | 12 | 1,757,121 | 6.83 |
|  | ACC | 16 | 2,539,804 | 6.30 |
|  | GGG | 12 | 1,982,325 | 6.05 |
|  | CCT | 15 | 2,725,243 | 5.50 |
|  | CTG | 13 | 2,491,907 | 5.22 |
|  | GTC | 10 | 2,072,510 | 4.83 |
|  | CCA | 17 | 3,531,944 | 4.81 |
|  | GTT | 19 | 3,980,510 | 4.77 |
|  | CAT | 20 | 4,419,148 | 4.53 |
|  | TGG | 16 | 3,519,990 | 4.55 |
|  | GGT | 11 | 2,530,602 | 4.35 |
|  | TCC | 12 | 2,923,974 | 4.10 |
|  | GGA | 12 | 2,917,986 | 4.11 |
|  | CCC | 8 | 1,975,774 | 4.05 |
|  | GCC | 7 | 1,757,558 | 3.98 |
|  | CTC | 12 | 3,063,515 | 3.92 |
|  | CTT | 18 | 4,686,490 | 3.84 |
|  | TTC | 18 | 4,804,696 | 3.75 |
|  | CAC | 10 | 2,670,848 | 3.74 |
|  | GCT | 9 | 2,466,905 | 3.65 |
|  | GAA | 17 | 4,796,876 | 3.54 |
|  | GCA | 10 | 2,854,157 | 3.50 |
|  | GTG | 9 | 2,668,711 | 3.37 |
|  | TCA | 15 | 4,543,623 | 3.30 |
|  | TCT | 14 | 4,517,008 | 3.10 |
|  | TGT | 13 | 4,193,986 | 3.10 |
|  | TAA | 16 | 5,198,741 | 3.08 |
|  | TTG | 17 | 5,535,900 | 3.07 |
|  | AGG | 8 | 2,721,537 | 2.94 |
|  | AGC | 7 | 2,466,223 | 2.84 |
|  | GAG | 8 | 3,062,341 | 2.61 |
|  | ATC | 9 | 3,501,207 | 2.57 |
|  | AAC | 10 | 3,997,880 | 2.50 |
|  | TGC | 7 | 2,851,016 | 2.46 |
|  | ACA | 10 | 4,194,082 | 2.38 |
|  | CTA | 6 | 2,787,091 | 2.15 |
|  | ACT | 7 | 3,319,865 | 2.11 |
|  | TTT | 20 | 9,584,339 | 2.09 |
|  | TGA | 9 | 4,545,032 | 1.98 |
|  | GTA | 5 | 2,634,595 | 1.90 |
|  | AGT | 6 | 3,311,685 | 1.81 |
|  | TAG | 5 | 2,777,280 | 1.80 |
|  | TTA | 9 | 5,197,586 | 1.73 |
|  | ATA | 9 | 5,459,641 | 1.65 |
|  | AGA | 7 | 4,502,957 | 1.55 |
|  | AAG | 7 | 4,668,999 | 1.50 |
|  | ATT | 10 | 6,871,128 | 1.46 |
|  | ATG | 6 | 4,413,505 | 1.36 |
|  | AAT | 9 | 6,868,709 | 1.31 |
|  | CAA | 6 | 5,540,023 | 1.08 |
|  | AAA | 8 | 9,541,736 | 0.84 |
|  | TAC | 2 | 2,643,798 | 0.76 |
|  | TAT | 4 | 5,459,104 | 0.73 |
|  | GAT | 2 | 3,498,505 | 0.57 |
