# Supplementary material for: Mutation rate analysis via parent–progeny sequencing of the perennial peach. I. A low rate in woody perennials and a higher mutagenicity in hybrids
Source: Proc Biol Sci. 2016 Oct 26;283(1841):20161016. doi: 10.1098/rspb.2016.1016 (PMC5095371; doi:10.1098/rspb.2016.1016)
Supplement: Supplementary Methods [file rspb20161016supp2.docx]

Supplementary methods for:

**Mutation rate analysis via parent-progeny sequencing of the perennial peach I. A low rate in woody perennials and a higher mutagenicity in hybrids**

Zhengqing Xie, Long Wang, Lirong Wang, Zhiqiang Wang, Zhenhua Lu, Dacheng Tian, Sihai Yang, Laurence D. Hurst

DOI: 10.1098/rspb.2016.1016

**1. Sampling**

For intraspecific group (group I), one F_1_ individual was obtained from a cross between two peach (*Prunus persica*) varieties in 2006, and was selfed in 2014 to generate the F_2_ fruits (Supplemental Fig S2). The F_2_ fruits were stored at low temperature for about three months to increase germination rate. Successful germinated seeds were then grown into small trees, and leaves from 24 randomly selected F_2_ saplings (144F2-1 to -24) were sampled three months later in 2015 (Supplemental Fig S2). The parent tree was about nine years old when sampled in 2015 with one arbitrary branch being chosen to collect fresh leaves for DNA extraction.

The above intraspecific group has a young F_1_ parent and a young F_2_ progeny. To attempt to exclude a possible influence of somatic mutations on our estimation, we additionally examined an intraspecific cross with a very old parent and a young F_2_ progeny. This intraspecific cross (group II) was a wild peach (*Prunus mira* Koehne) sampled in Nyingchi, Tibet, China (latitude 29°42.018' N, longitude 94°20.560' E). The F_1_ parent tree has been growing for approximately 200~300 years. The tree was over eight meters tall and with a trunk over one meter in diameter. The tree was geographically isolated from other wild peach trees, and the fruits from this tree were assumed to be from selfing. It is unknown whether this parent tree itself was from selfing or outcrossing of different *P. mira* trees. This tree was sampled in May 2015, prior to that year’s seed set. The F_2_ seeds were collected under this tree in 2014. The sequencing data confirmed that the progeny were from selfing, as no other genetic source has been observed excepting the parent tree. Some of the F_2_ seeds were then treated with Gibberellin (GA) to enable fast germination (requiring only 1~2 weeks). Eight successfully germinated F_2_ individuals (GZTH-S1 to –S5, -S7 to –S9) were grown in a light incubator for about 2~3 months for leaf sampling (Supplemental Table S1). Two samples (GZTH-5 and GZTH-8) were not germinated and the seed was directly used as raw material after carefully removing the seed coat. One sample GZTH-8 was highly contaminated and sequenced with low quality. The sequencing data from this sample was thus only used to enable removal of false positive mutation calls.

The interspecific F_1_ (group III) was a successful cross between the domesticated peach and its wild relative (*Prunus davidiana*) in 2005 (Supplemental Fig S2). The F_1_ individual was selfed in 2010 to derive F_2_ individuals. The pedigree to derive the F_1_ individual is described in Supplemental Fig S1. The F_2_ fruits were treated with low temperature storage for about one year and grown into small tress after successful germination (Supplemental Fig S2). Those F_2_ seeds were grown for about three years from 2012 to 2015 until sampling (Supplemental Fig S2). The parent tree was also sampled in 2015 with an age of about 10 years old. Leaves for the F_1_ parent tree and four of its ancestors as well as 30 of its progeny (Supplemental Table S1) were collected from an arbitrary branch of each individual. Note that the age of the F_1_ parental trees in crosses I and III are approximately the same, while that of cross II is much older. Should somatic mutations be causing a major difference between crosses I and III, the effect should be even more profound in cross II.

All of the F_1_ and F_2_ samples from group I and III were preserved in the National Fruit Tree Germplasm Repository, Zhengzhou Fruit Research Institute, Chinese Academy of Agricultural Sciences, China (Supplemental Table S1).

**2. Estimation of callable sites and false negative rate**

To estimate the number of callable sites and the false negative rate we employed a simulation method. The empirical read-depth distribution for each group was sampled from F_2_s within sites with a confident heterozygous genotype in F_1_ parent (maker sites used in detecting recombinant blocks). The synthetic mutations were generated from all F_2_s using a custom PERL script sim_mutation_reads.pl (<https://github.com/wl13/BioPipelines/>tree/master/Mutation_Simulation) with options “--random-size 1000 --samtools ‘-F 3844’ --exclude scaffold”. The samtools flag ignores those unmapped or supplementary reads that were not informative. A total of 1,000 synthetic mutations were generated on eight chromosomes for each group (Supplemental Table S2). These edited reads were then processed with the same pipelines to detect the synthetic mutations. The fraction of callable simulated mutated sites was used to estimate the fraction of callable sites in the genome for each group (Supplemental Table S2). Uncallable sites include sites that could not be called by any callers (e.g. due to low mapping quality), sites with a low-depth (<5) or strand bias (only have forward or reverse strands) in the mutated sample, and sites not informative in parental samples or other F_2_s (Supplemental Table S2). Among all callable sites, a total of 15 simulated sites failed to be recovered. Seven sites were due to high sequencing errors in other samples (>4 samples) or putative contamination in another sample, which happened to have an allele identical to the synthetic allele. Another 10 sites were found to reside in homopolymer or tandem repeat regions which also have indels nearby. These latter sites were actually captured as indel mutations and could be recovered through manual investigation.

**3. Estimation of diversity levels**

The population diversity was calculated as the average pairwise differences among all possible pairs. The pairwise difference was defined as the per site nucleotide difference between each of the two compared individuals, e.g. 1 would be counted for a difference between two different homozygous genotypes while 0.5 would be counted for a difference between a homozygous genotype and a heterozygous genotype. The pairwise differences were obtained by first summing up all nucleotide differences in a window, then dividing by the number of informative sites (sites genotyped in both individuals) in the same window. For each pair, only windows with more than 50% informative sites were considered as an informative pair in this window. Windows with less than 1208 informative pairs (i.e. 50% of all total 2415 pairs) were discarded from the correlation test.

**4. Removing pseudo-heterozygous sites**

Mapping errors due to highly similar paralogous sequences could also result in pseudo-heterozygosity. To minimize these errors, we remove those markers residing in large structural variant (SV) regions of F_1_ samples compared to the reference genome in each group. The SVs were detected by combining three different algorithms: a read-depth approach (CNVnator) [47], a split-read approach (Pindel) [48] and from the analysis of discordant pairs (Breakdancer) [49]. CNVnator (version 0.3) was run with a bin size of 100bp, which predicts large deletions and duplications. Pindel (version 0.2.5b6) was run with default options. Results were collected for large deletions (≥ 100bp), inversions, and translocations. Deletion, duplication and inversion results were also collected from Breakdancer (version 1.1.2) with default settings. We generated a union set of results collected from all three approaches without further filtering. SVs with a size smaller than 100kbp were directly used. We also include 200bp flanking regions of all inversion events. For SVs larger than 100kbp, we use the 400bp flanking regions around each predicted SV breakpoint.
